# Did the Family Health Strategy have an impact on indicators of hospitalizations for stroke and heart failure? Longitudinal study in Brazil: 1998-2013

**DOI:** 10.1371/journal.pone.0198428

**Published:** 2018-06-26

**Authors:** Denise de Fátima Barros Cavalcante, Valéria Silva Cândido Brizon, Livia Fernandes Probst, Marcelo de Castro Meneghim, Antonio Carlos Pereira, Gláucia Maria Bovi Ambrosano

**Affiliations:** Department of Community Dentistry, Piracicaba Dental School, University of Campinas (UNICAMP), Piracicaba, SP, Brazil; Leibniz Institute for Prevention Research and Epidemiology BIPS, GERMANY

## Abstract

**Introduction:**

The objective was to analyze whether socioeconomic factors related to the context and those related to the model of care—specifically the coverage of primary care by the Family Health Strategy (ESF)—had an impact on hospitalizations due to heart failure (HF) and stroke, in the State of São Paulo/Brazil between 1998 and 2013.

**Methods:**

A longitudinal ecological study involving 645 municipalities was conducted in the state of São Paulo/Brazil from 1998 to 2013, using the Hospital Information System (SIH–DataSUS database). The hospitalizations for primary care sensitive conditions: Stroke and heart failure (HF) that correspond to the International Classification of Diseases (ICD 10): I50, I63 to I67; I69, G45 to G46 were analyzed longitudinally during the period indicated regarding the percentage of people covered by the Family Health Program (PSF) adjusted for confounders (population size, gross domestic product -GDP and human development index- HDI).

**Results:**

There was a significant decrease in the number of hospitalizations for heart failure and stroke per 10000 (inhabitants) in the period (p <0.0001), with a significant relationship with increased proportion of ESF (p <0.0001), and this remained significant even when possible confounders (population size, GDP and HDI) were included in the model (p <0.0001).

**Conclusions:**

GDP per capita was close to or higher than that if many European countries, which shows the relevance of the study. The health care model based on the Family Health Strategy positively impacted hospitalization indicators for heart failure and stroke, indicating that this model is effective in the prevention of primary care sensitive conditions.

## Introduction

Ambulatory or primary care sensitive conditions (ACSC) represent a set of indicators used internationally to indicate potentially preventable diseases by access to and quality of Primary Health Care. Access to services, continuity of care and the effectiveness of actions in primary care, by avoiding hospitalization and deterioration of the problems, as opposed to high hospitalization rates, reflect deficiencies in the coverage and performance of primary health care (PHC) [1–3].

The implementation of an indicator for primary care sensitive conditions (PCSCs) may have different purposes, following changes in the health systems used. In the United States, hospitalizations for PCSCs were used to evaluate the performance of health systems, reflecting the accessibility of health services [4]. For example, in the UK, where access to health care is universal, as it is in Brazil, this measurement has become of interest to measure the quality of services offered [5].

In Brazil, with the Health Pact in 2006, health indicators were established for the Unified Health System (SUS) [6] and later the Brazilian List of Hospitalization for Primary Sensitive Conditions was published [7]. Nowadays managers can use this list and take it as reference to the impact that major health disorders could cause in the number of hospitalizations, because previously there were no parameters set for this purpose [6,7].

The Family Health Strategy (ESF) can be considered the government's main efforts to improve primary health care in Brazil. The ESF offers a wide range of primary health care services provided by a team consisting of one doctor, one nurse, one nursing assistant, and four or more community health workers. Some teams also include an oral health team (dentist and assistant). Each team is assigned to a geographic area that is responsible for registering and monitoring the health status of the population in this area, providing primary care services, and making referrals to other levels of care as needed. Each team is responsible for an average of 3450 and a maximum of 4500 people [8]. In 2013 the population coverage of the ESF and the PACS (Community Health Workers Program) was 53.37% and 64.74%, respectively [9]. Even with government´s efforts with financial incentives to increase the number of ESF teams and ACS, there is still little scientific evidence of the impact of primary care on health indicators policies in Brazil.

The intervention in primary care sensitive diseases makes it possible to improve the indicators of processes (evidence-based decisions, regulatory services, access) and results (user satisfaction, improved indicators, cost reduction) [3]. An effective approach to health care must target a specific level of quality that improves access to health care strategies to strengthen the family and community to cope with and control infectious diseases through self-care and health protection [10,11].

Although a few national studies have indicated a decline in hospitalization rates for heart failure and stroke in both sexes [3,12], cardiovascular diseases are the leading cause of death in the world, and in essence, they lead to a high social and economic burden; where the deaths represent 20% of the cases in high-income countries, 80% of them occur in low- and middle-income countries [13,14].

Thus, due to the worldwide expressiveness of these two indicators (CI and stroke) and with the purpose of contributing to the study of how the cited indicators have performed over time, in the presence of a universal health program, this study aimed to longitudinally assess the impact of PSF on the indicators of hospitalizations for primary care sensitive conditions: heart failure (HF) and cerebrovascular accident (CVA) in the municipalities of the state of São Paulo/Brazil from 1998 to 2013.

## Methods

The Research Ethics Committee of Piracicaba School of Dentistry—UNICAMP approved the study (Protocol No. 013/2016). This study with a longitudinal and ecological design assessed how the population behaved on a collective, not individual, and longitudinal level [15]. The state of São Paulo had 645 representative units (municipalities), with a population of 43,663,669 inhabitants [16] in 2013. We evaluated the impact of the Family Health Strategy (ESF) on the number of hospitalizations for heart failure (HF) and stroke per 10,000 inhabitants from 1998 to 2013.

The causes 11 and 12 of constant admissions in the ordinance 221/2008 [7] of the Ministry of Health were considered Primary Care Sensitive Conditions (PCSCs). These causes are identified by International Classification of Diseases (ICD-10) such as heart failure (I50) and cerebrovascular diseases (I63 to I67, I69, G45 and G46) [17].

The dependent variables were the number of hospitalizations for "Heart failure (HF) and stroke" in both sex and all ages—both data obtained from the Hospital Information System (SIH). These indicators are obtained by the municipality, and are arranged in aggregate, not individualized form, provided by DATASUS [18]. The SIH provides information on admissions to public and private hospitals of the Unified System of Health from Brazil (SUS). The independent variables considered were: period of time (in months) from the first implementation of ESF- (Department of Primary Care—DAB) [9], the proportion of municipal population covered by the ESF (DAB) [9], period of time (in months) from the last implementation of the ESF (DAB) [9], population (IBGE) [15], GDP (Gross Domestic Product) [19] and mHDI- Municipal Human Development Index (SEADE Foundation-SP) [19].

### Data analysis

Initially descriptive statistics of the variables were calculated. Later regression models were adjusted to assess the association of ESF and ACS coverage ratio (all ACS irrespective of the care model) with the number of admissions for HF and stroke. As these data consisted of repeated measurements, the presence of correlations among observations of the same municipalities was assumed. Thus, the data were analyzed using Generalized Linear Mixed Models for Non-Gaussian Longitudinal Data using GLIMMIX of the SAS [20]. The models were initially estimated considering the number of hospitalizations for HF and stroke as dependent variables and the proportion of ESF and ACS (adjusted by year) as predictors. The next step was to test the possible confounding variables (population, GDP and mHDI) in the model.

## Results

Table 1 shows the data of descriptive statistics related to the number of hospitalizations for heart failure (HF) and stroke (per 10,000 inhabitants.) in the state of São Paulo between 1998 and 2013. HF has decreased over the years from a median value of 26.9/10,000 inhabitants in 1998 to 11.7/10,000 inhabitants in 2013. The stroke values showed fluctuations over time, presenting median values of 6.2/10,000 inhabitants in 1998, 10.3/ 10,000 inhabitants in 2004 and 6.5/10,000 inhabitants in 2013.

**Table 1:** Median (minimum and maximum) number of hospitalizations for heart failure and strokes for 10,000 inhabitants in the cities of São Paulo due time.

Year	Heart Failure	Stroke
1998	26.9 (0.05–210.7)	6.2 (0.04–312.7)
1999	27.8 (0.2–178.0)	8.9 (0.07–382.4)
2000	24.9 (0.6–231.0)	9.3 (0.06–409.0)
2001	23.5 (0.6–257.6)	10.0 (0.1–295.1)
2002	22.6 (0.1–209.0)	9.7 (0.2–236.4)
2003	21.6 (0.6–148.4)	9.8 (0.1–262.4)
2004	20.9 (0.7–205.8)	10.3 (0.1–177.7)
2005	19.3 (0.6–205.8)	8.6 (0.03–174.1)
2006	18.4 (0.2–156.3)	9.0 (0.09–185.8)
2007	17.4(0.09–145.1)	8.0 (0.04–218.7)
2008	14.5 (0.5–207.0)	5.9 (0.04–218.7)
2009	15.5 (0.3–329.9)	7.0 (0.2–165.1)
2010	15.1 (0.3–269.3)	7.5 (0.06–126.4)
2011	14.7 (0.3–267.2)	8.1 (0.2–141.1)
2012	12.9 (0.6–279.2)	7.9 (0.09–113.2)
2013	11.7 (0.5–259.5)	6.5 (0.1–63.7)

Table 2 shows the median value of the proportion of the ESF and PACS between 1998 and 2013 in the state of São Paulo. Proportion of ESF remained with a median of 0% between 1998 and 2000, and increased from 2001 to 2013, ranging from 4.9 to 40.2% coverage. Moreover, the population coverage from 25 thousand to over 31 thousand could be seen. GDP ranged from R$5494.38 to R$18620.84, while the HDI ranged from 0.788 to 0.688 from 1998 to 1999; and (due to index metric questions) remained at this level until 2009, and increased to 0.729 in 2012–2013.

**Table 2:** Median (minimum and maximum) of the proportion of the Family Health Strategy (ESF), Health Program of Community Agents (PACS), population, Gross Domestic Product (GDP) and the Human Development Index (HDI) in the cities of São Paulo according to the time.

Year	ESF%	PACS%	Population	GDP	HDI
1998	0 (0–77.6)	0 (0–80.1)	25068.0 (1961.0–9887614.0)	-	0.788 (0.5–0.8)
1999	0 (0–100.0)	0 (0–100.0)	25068.0 (1961.0–9887614.0)	5494.9 (1868.2–56985.2)	0.688 (0.5–0.8)
2000	0 (0–100.0)	5.3 (0–100.0)	26085.0 (1905.0–9968485.0)	5770.5 (2049.7–93963.7)	0.688 (0.5–0.8)
2001	4.9 (0–100.0)	11.6 (0–100.0)	27891.0 (1836.0–10499133.0)	6610.3 (2235.1–88847.4)	0.688 (0.5–0.8)
2002	10.3 (0–100.0)	20.2 (0–100.0)	27891.0 (1836.0–10499133.0)	7673.4 (2466.2–107008.0)	0.688 (0.5–0.8)
2003	13.8 (0–100.0)	24.7 (0–100.0)	28174.0 (1795.0–10600060.0)	8566.4 (2705.7–109963.1)	0.688 (0.5–0.8)
2004	19.0 (0–100.0)	28.8 (0–100.0)	28726.0 (1749.0–10677019.0)	8934.5 (2763.8–101877.1)	0.688 (0.5–0.8)
2005	21.7 (0–100.0)	31.2 (0–100.0)	28726.0 (1749.0–10677019.0)	9561.8 (3135.0–102099.9)	0.688 (0.5–0.8)
2006	25.0 (0–100.0)	34.8 (0–100.0)	30159.0 (1599.0–10927985.0)	10719.9 (3343.4–138980.8)	0.688 (0.5–0.8)
2007	27.4 (0–100.0)	36.9 (0–100.0)	30384.5 (1546.0–11016703.0)	12043.3 (4282.2–211883.8)	0.688 (0.5–0.8)
2008	32.2 (0–100.0)	48.3 (0–100.0)	30384.5 (1546.0–11016703.0)	12357.9 (4736.3–171506.5)	0.688 (0.5–0.8)
2009	33.0 (0–100.0)	46.0 (0–100.0)	29817.5 (1663.0–11168194.0)	14224.4 (5672.5–163436.4)	0.689 (0.5–0.8)
2010	38.1 (0–100.0)	47.3 (0–100.0)	30066.0 (1643.0–11245983.0)	16588.6 (6285.1–241014.6)	0.729 (0.6–0.9)
2011	38.5 (0–100.0)	50.4 (0–100.0)	30290.0 (1627.0–11312351.0)	17752.6 (6743.5–287501.3)	0.729 (0.6–0.9)
2012	40.3 (0–100.0)	56.3 (0–100.0)	30603.0 (1612.0–11379114.0)	18620.8 (7232.6–283589.5)	0.729 (0.6–0.9)
2013	40.2 (0–100.0)	52.6 (0–100.0)	31063.5 (2856.0–11446275.0)	18620.8 (7232.6–283589.5)	0.729 (0.6–0.9)

Tables 3 and 4 show the results of estimated regression models. There was a significant decrease in the number of hospitalizations for HF (p< 0.0001). A significant relationship was also observed between the number of hospitalizations for HF and stroke per 10,000 inhabitants with the increase in ESF proportion (p <0.01) (model 1) and this relationship remained significant when possible confounders (population, GDP and HDI) were included in the model (p <0.001) (model 2).

**Table 3:** Generalized linear mixedmodels of hospitalizations for heart failure per 10,000 inhabitants.

	Model 1 (Unadjusted)			Model 2 (^$^adjusted)		
Variable	Estimate	Standard Error	p-value	Estimate	Standard Error	p-value
Year	-0.04598	0.001696	<0.0001	-0.04894	0.002503	<0.0001
ESF proportion	-0.00156	0.000542	0.0041	-0.00181	0.000570	0.0015
ACS proportion	-0.00084	0.000519	0.1055	-0.00070	0.000545	0.2004

Note: ESF: Family Health Strategy, ACS: Health of Community Agents.

^$^ adjusted for Population, GDP (Gross Domestic Product) and HDI value (Human Development Index).

**Table 4:** Generalized linear mixedmodels of hospitalizations for stroke per 10,000 inhabitants.

	Model 1 (Unadjusted)			Model 2 (^$^adjusted)		
Variable	Estimate	Standard Error	p-value	Estimate	Standard Error	p-value
Year	-0.01475	0.00246	<0.0001	-0.02614	0.003554	<0.0001
ESF proportion	-0.00413	0.000762	<0.0001	-0.00407	0.00079	<0.0001
ACS proportion	-0.00139	0.00072	0.0521	-0.000744	0.000746	0.3185

Note: ESF: Family Health Strategy, ACS: Health of Community Agents.

^$^ adjusted for Population, GDP (Gross Domestic Product) and HDI value (Human Development Index).

## Discussion

The analysis indicated that there was significant decrease in the number of hospitalizations for HF in the state of São Paulo in the period 1998–2013 (p <0.0001). The number of hospitalizations for heart failure and strokes was associated with the increase in the Family Health Strategy proportion (p <0.01) and this finding remained even with inclusion of potential confounding covariates in the model (population, GDP and HDI). However, the coefficients were low, since the magnitude of the effects was small. Nevertheless, the data suggested effectiveness of the primary care approach in prevention of PCSCs.

Brazilian National Primary Care Policy includes several associated factors that may have contributed simultaneously to the decrease in hospitalizations such as the longitudinal patient care approach through the offer of multidisciplinary teams, free therapeutic support and preventive policies and treatment protocols [21–24]. All these factors are also mentioned in the Strategic Action Plan for Confronting Noncommunicable Chronic Diseases (CNCD) from 2011 to 2022 [25].

In the period of this study several health policies were in force or were implemented, which may have been responsible for the reduction in indicators during this time.

Evaluating ESF in Brazil, with a longitudinal approach in 30% of the municipalities in Brazil, showed that population coverage was directly associated with the reduction in hospitalizations and mortality rates due to cerebrovascular disease, and its effect increased within the implementation period [26]. Another study found recurrence of cardiovascular disease in primary health care models with and without ESF, both models of state care in Brazil. The model with ESF was associated with lower risk of death from all causes, and there was a 16.4% reduction in the absolute risk of death from cardiovascular disease for ESF [27]. This last previously published study corroborated the findings of a cohort study conducted in Paris, which examined the increased incidence of cardiovascular diseases as factors related to the social determinants and decrease in services [28].

In addition to access to and quality of ESF care, preventive and health promotion initiatives, such as the implementation of Centers to Support Family Health (NASF) occurred simultaneously in several municipalities. These centers offered differentiated collective action services, with a multidisciplinary team approach including incentives to reduce smoking, alcoholism and encouraging healthy practices—for example the abandonment of inactivity [29].

The lack of access to medicines in the public sector is one of the major barriers to fighting the chronic diseases, and management models should intensify efforts to adopt public policies that guarantee dispensed medication [30]. Thus, in Brazil, the use of therapeutic protocols by the National Medication Policy [31] (1998) and the National Policy on Pharmaceutical Care [32] (2004) has ensured that many essential medicines are available free of charge, or at a reduced cost [33], thereby enhancing the care and strengthening the assistance.

Another protective factor related to the reduction in hospitalizations was the implementation of an immunization policy against influenza since 1999. This was important because patients infected with influenza and respiratory infections showed a hemodynamic instability, and immunization against influenza was a protective factor in preventing morbidity and mortality from cardiovascular disease [34].

Since 1980, tobacco control that includes a set of actions to reduce the prevalence of smoking has been articulated by the Brazilian Ministry of Health. However, only in 2003 Brazil signed the Framework Convention on Tobacco Control (FCTC) with the effective implementation of actions between 2005 and 2006. It was the most relevant event that resulted in the implementation of National Policy to Control Tobacco Use with actions focused on the reduced demand of tobacco, with an important market regulation, protection measures such as banning smoking in collective environments and promoting cessation of tobacco use [35,36]. This approach corroborated the findings of a previous study that showed a strong association of chronic conditions with major cardiovascular risk factors for smoking [37].

Another transversal policy was the development and implementation of the Strategic Action Plan for Confronting Chronic Noncommunicable Diseases (2011–2022), with nine national targets related to morbidity and mortality issues and four risk factors: smoking, inadequate diet, physical inactivity, and excessive use of alcohol; and four groups of lesions: cardiovascular, cancers, diabetes and chronic respiratory diseases [38,39].

Analyzing the ranking of the major causes for Disability-Adjusted Life Year (DALY) in Brazil and macro-regions (data not shown), it was shown that for Brazil, as a whole, diabetes mellitus (5.1%), ischemic heart disease (5.0%) and stroke—first occurrence (4.6%), totalizing 14.7% of the total DALYs was characteristic of an epidemiological pattern of developed countries [40]. Moreover, in the WHO survey conducted in 23 countries, including Brazil, losses of $ 84 billion due to coronary heart disease, stroke and diabetes were estimated from 2006 to 2015 [41].

Cardiovascular diseases, despite their decline, have been and continue to be the main cause of death in Brazil. The decline in cardiovascular disease was greater for cerebrovascular diseases (34%) and heart disease (44%). Mortality from ischemic heart disease decreased by 26% [29].

As differential, this study presented a longitudinal analysis, as there are few in the literature, especially using mixed models to verify the impact of the health care policy over time. It is worth noting that the ESF has been present since 1994, but the state of São Paulo was the last state to implement the strategy, although it is the state with the highest HDI and largest population (over 40 million). Despite the Strategy´s own funding, with financial incentives for incorporating family health teams, oral health teams, NASF teams and other initiatives, there was still a delay in implementing this strategy, which made it difficult to change the health care policy, as demonstrated by the slow growth in population coverage from 1998 to 2013 (which made the results more interesting), far below the percentage of other regions of Brazil. Nevertheless, the statistical model was able to identify changes in the epidemiological profile and its relationship with the population covered by the ESF.

We emphasize this was an ecological study and limitations were expected. The first is the possibility of ecological fallacy, in which ecological associations do not always reflect individual associations. It was not possible to determine whether Individuals with the outcomes were under ESF coverage, because the level of aggregate was the municipality [26].

Furthermore, the ESF was influenced by political changes. Changes of healthcare managers, since they have their political ideologies and personal conceptions of public management, and these did not always coincide with the current Brazilian health policy. Nevertheless, two English studies have confirmed that the provision of care in Primary Care had a direct impact on mortality rates and recommended that improvement in the health of the population required a reduction in health inequalities; and it was treated as a political priority with effective territorial monitoring for reduction of hospitalizations and consequently of mortality [42,43].

Finally, we could assume that the result for the state of São Paulo could be inferred for all other states in Brazil, considering that the beginning and later development of this strategy in São Paulo was very precarious and presented numerous technical difficulties, mainly because the State had an organized primary health care network in the 1990s.

## Conclusion

We concluded that the health care model based on the Family Health Strategy has positively impacted the hospitalization indicators for heart failure and stroke, indicating that this model was effective in preventing Ambulatory or primary care sensitive conditions (PCSCs).

## Supporting information

S1 File Study data. (XLS)
